# Physical presence of spouse enhances brain-to-brain synchrony in co-parenting couples

**DOI:** 10.1038/s41598-020-63596-2

**Published:** 2020-05-05

**Authors:** Atiqah Azhari, Mengyu Lim, Andrea Bizzego, Giulio Gabrieli, Marc H. Bornstein, Gianluca Esposito

**Affiliations:** 1 Psychology Program, School of Social Sciences, Nanyang Technological University, Singapore, Singapore; 2 Division of Psychology, Department of Psychology and Cognitive Science, University of Trento, Trento, Italy; 3 National Institute of Child Health and Human Development, Bethesda, USA; 4 Institute for Fiscal Studies, London, United Kingdom; 5 UNICEF, New York, USA; 6 Lee Kong Chian School of Medicine, Nanyang Technological University, Singapore, Singapore

**Keywords:** Social behaviour, Cooperation, Human behaviour

## Abstract

Co-parenting spouses who live together remain in close physical proximity to each other and regularly engage in reciprocal social interactions in joint endeavors to coordinate their caregiving. Although bi-parental rearing is a common occurrence in humans, the influence of the physical presence of a co-parenting spouse on parental brain responses remains largely unknown. Synchrony is conceptualized as the matching of behavioral and physiological signals between two individuals. In this study, we examined how the presence of a co-parenting spouse influences brain-to-brain synchrony when attending to salient infant and adult vocalizations. We hypothesized that brain-to-brain synchrony would be greater in the presence of a spousal partner. Functional Near-infrared Spectroscopy (fNIRS) was used on 24 mother-father dyads (N = 48) to measure prefrontal cortical (PFC) activities while they listened to infant and adult vocalizations in two conditions, together (in the same room at the same time) and separately (in different rooms at different times). Couples showed greater synchrony in the together condition; when comparing fNIRS data between true couples and randomly matched controls, this synchronous effect was only seen in true couples, indicating a unique effect of spousal co-regulation toward salient stimuli. Our results indicate that the physical presence of the spouse might establish synchrony in attentional regulation mechanisms toward socially relevant stimuli. This finding holds implications for the role of the co-parenting spouse in influencing social and parental brain mechanisms.

## Introduction

Parturition in humans initiates collaborative efforts between spouses toward effective co-parenting^1^. Although an evolutionary perspective asserts specific parenting roles of mothers and fathers^2^, the transition to parenthood in bi-parental species is accompanied by parallel modifications in mothers’ and fathers’ brains, notably in regions implicated in attention, cognition, and affect regulation^2–4^. Thus, the emergence of co-parenting in humans is likely supported by biobehavioural synchrony in couples^3,5^, which entails the entrained temporal coordination of physiological and behavioural signals between two individuals in an affiliative bond^6^. For co-parenting couples, constant interaction with each other presents partners with abundant opportunities to engage in daily rhythms of reciprocal exchanges that establish synchrony across numerous biological systems^7^. For example, couples have been shown to exhibit synchronised patterns of gaze and affect along with coordinated changes in electrodermal activity^8^, respiratory sinus arrhythmia^9^, diurnal cortisol patterns^10^, and brain activation patterns^11,12^. Because synchrony is associated with enhanced mutual attunement to emotional states^13^ and facilitates behavioral and physiological coordination between partners^14,15^, theories have been advanced that such synchrony may constitute a central pathway to achieving emotional stability^16,17^. When partners are “in-sync” in their subjective emotional experiences, they respond more optimally to one another’s needs and bolster support for one another^14^. Partner support buffers stress evoked by the novel demands of parenting and forms a critical ingredient in adaptation to parenthood^18,19^.

The pursuit of a joint goal to protect the altricial human infant provides fertile ground for adaptive and synchronous functioning of mothers’ and fathers’ brains^20^. To our knowledge, only one study has so far investigated co-parenting and brain-to-brain synchrony: Using fMRI imaging techniques, the authors examined co-parents’ brain responses to viewing own infant play videos in comparison with standard infant play videos. Results showed that synchrony in neural circuits involved in empathy and social cognition emerged in co-parents exposed to infant distress cues^3^. This finding suggests that mothers and fathers attune their brain responses to each other and that brain-to-brain synchrony may stem from unique couple coordination. However, the circumstance under which this coordination emerges requires further verification. Among studies that investigated physiological synchrony in couples, some have evinced the importance of physical presence of a spouse in initiating synchrony, whereas others have determined that synchrony is still observed in the absence of a spouse. A study that measured cortisol and mood patterns of spousal partners^21^ revealed that the extent to which biological rhythms of spousal partners synchronise hinges greatly on the amount of time the two spent in close proximity. When cortisol levels were compared at times when partners were together and apart, synchrony in cortisol level was evident only when couples were together. Conversely, another study^22^ found that synchrony in cortisol level was not dependent on whether the spousal partner was present, but was instead determined by the amount of time spousal partners spent with each other throughout the duration of the experiment. Taken together, these contradictory findings underscore an important gap in our knowledge regarding whether the physical presence of a spousal partner affects dyadic synchrony. Understanding this association may aid in uncovering how spousal proximity in day-to-day life facilitates efficient co-parenting responses.

To fill this research gap, the present study employed functional Near-infrared Spectroscopy (fNIRS) to measure the brain signals of co-parents when they were exposed to salient infant and adult vocalisations either together (in the same room at the same time) or separately (in different rooms at different times). We decided to focus on the prefrontal cortical (PFC) region of the brain due to its integral role in both attentional regulation and social cognition, making it a likely region to be implicated when partners jointly attend to salient emotive vocalisations^23–26^. First, we aimed to investigate whether synchrony was significantly higher when couples were in the physical presence of one another compared to when they listened to vocalisations separately. To do so, we derived a synchrony index. As the co-parents in our sample lived in the same household, we hypothesised that, compared to when they listened to salient vocalisations in the absence of their partner (separate-condition; SEP), couples would exhibit greater brain-to-brain synchrony in the physical presence of each other (together-condition; TOG)^21^. Second, to ascertain whether the co-presence effect was a result of the physical presence of a co-parent, or due to the unique pairing of couples who had existing relationships with each other, we compared the synchrony index of true couples to that of control couples (a synchrony index generated from randomly paired brain signals of a mother and father who were not spouses) in both the SEP and TOG conditions. We hypothesized that synchrony would only be observed between true couples but not in control couples. Third, we aimed to investigate whether stimuli- and parent-related factors play a role in driving the enhanced synchronous response observed in TOG compared to SEP. Thus, in channels where, compared to SEP, synchrony was found to be higher in TOG, we would examine if synchrony was influenced by emotional valence of acoustic stimuli (i.e. positive or negative vocalisations) and parents’ characteristics, namely, (i) the ratio of mother to father taking the lead in attending to the child, (ii) primiparous or multiparous status of parents, and (iii) age of parents. As little is known regarding the effects of these variables on brain-to-brain synchrony, we did not have any specific hypotheses for this third exploratory aim.

## Results

The first aim of the study was to verify that, compared to the separate-condition (SEP), synchrony was higher in the together-condition (TOG) condition for true dyads. Analysis of Variance (ANOVA) revealed that the difference in synchrony index between SEP and TOG was significant in four fNIRS channels, namely channel 3 (CH3), CH7, CH11 and CH13 (see Table 1), which were mapped to the left inferior frontal gyrus (IFG), left middle frontal gyrus (MFG) and left as well as right bilateral anterior PFC (aPFC), respectively. In all significant channels, the synchrony index in TOG was higher than in SEP (see Table 1).

**Table 1:** Mother-father synchrony indexes in true dyads.

Channel	Area	SEP			TOG			p (uncorrected)	p	d
		Mean	SD	N	Mean	SD	N			
3	IFG	0.007	0.025	141	0.031	0.083	138	0.00221	0.01471	0.4
7	MFG	0.009	0.025	135	0.024	0.045	132	0.00041	0.00409	0.4
11	aPFC	0.013	0.064	135	0.062	0.119	138	0.00003	0.00062	0.5
13	aPFC	0.026	0.109	135	0.043	0.090	138	0.00484	0.02421	0.2

Comparison between separate-condition (SEP) and together-condition (TOG) for the significant synchrony indexes. Note: IFG = inferior frontal gyrus, MFG = middle frontal gyrus, aPFC = anterior prefrontal cortex.

The second aim of the study was to prove that the co-presence effect was due to the unique physical presence of a co-parenting spouse. The same ANOVA analysis was conducted on control dyads but the differences in synchrony index between TOG and SEP were never significant in any channel (see Supplementary Table 1 for results for all channels). Taken together, significant differences between TOG and SEP in only true dyads, but not control dyads, confirmed that the unique presence of a spousal partner increases couple’s brain-to-brain synchrony.

The third aim of this study was to test whether the co-presence effect also depended on the type of acoustic stimuli and parent-related characteristics: (i) ratio of mother to father taking the lead in attending to the child which was measured by the Average Co-parenting Ratio Score, (ii) primiparous or multiparous parents, and (iii) age of parents.

### Synchrony and type of acoustic stimulus

**Figure 1:**
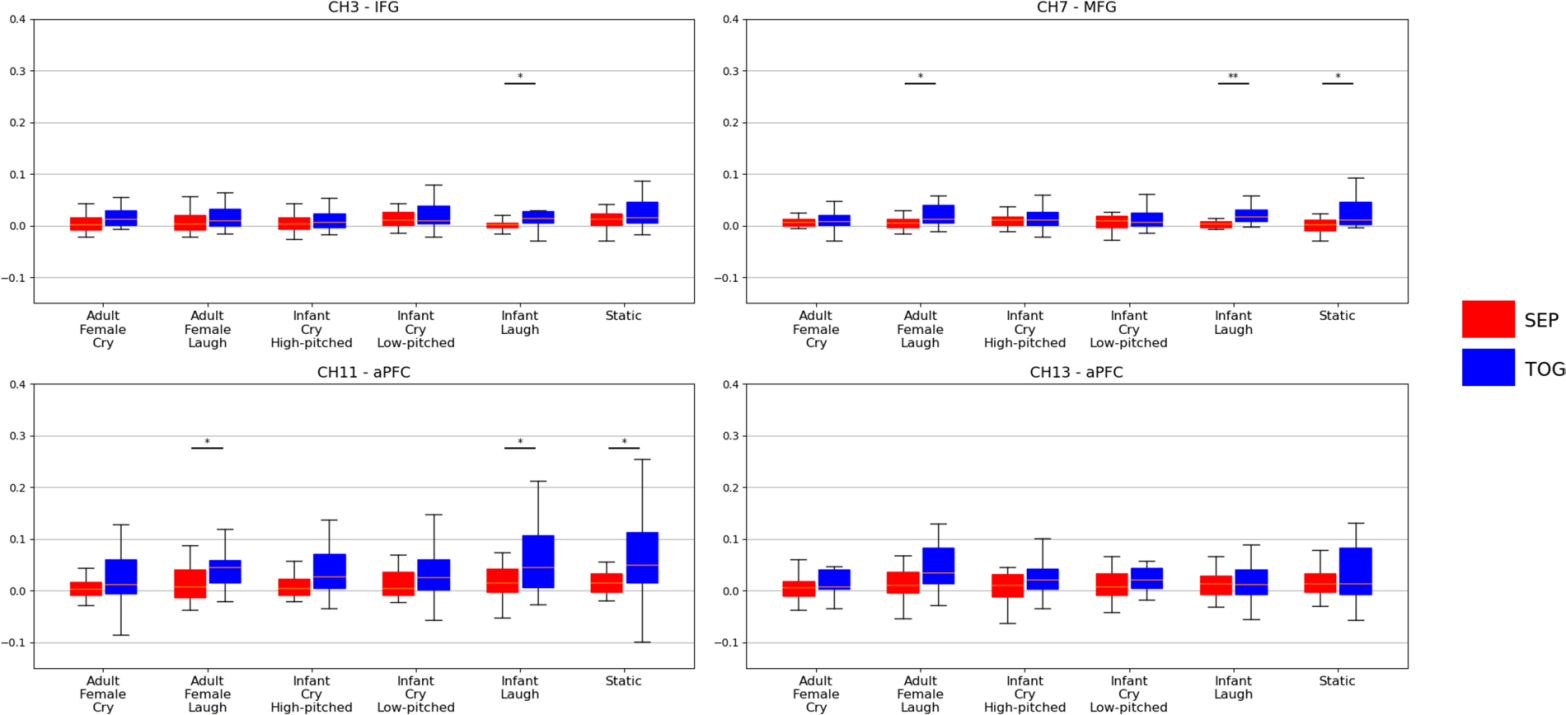
Comparison of the MCC2 measures between the SEP (red) and TOG (blue) conditions for the six stimuli in the four channels for which a significant effect of physical presence was found. Outliers are not shown.

Comparing the difference in synchrony indexes between SEP and TOG in the four significant channels, for each acoustic stimulus, we observed that infant laughter, adult laughter, and static sound induced greater synchrony in TOG compared to SEP (see Fig. 1). Except for CH13 (right aPFC), for which no statistical difference across acoustic stimuli was found, infant laughter significantly increased synchrony index in CH3 (left IFG), CH7 (left MFG) and CH11 (left aPFC), while adult laughter and static only increased synchrony in CH7 (left MFG) and CH11(left aPFC). Notably, differences for Infant Cry, both high- and low-pitched, as well as Adult Female Cry were never significant.

No other channel showed significant differences between SEP and TOG across the different acoustic stimuli.

### Synchrony and parent characteristics

First, synchrony was positively correlated with the Average Co-parenting Ratio Score, where a high ratio is associated with the predominance of the mother (rather than the father) in responding to the child (see Supplementary Table 2): *ρ* = 0.18, p = 0.00003; significant correlations between synchrony index and Average Co-parenting Ratio Score emerged for CH7 (MFG, *ρ* = 0.29, p = 0.004) and CH11 (left aPFC, *ρ* = 0.23, p = 0.015).

Second, significantly (p = 0.00066) higher synchrony was found for primiparous (Mean = 0.06, SD = 0.117) compared to multiparous parents (Mean = 0.028, SD = 0.065) when considering all channels together; and for Channel 7 (p = 0.036; Primiparous: Mean = 0.041, SD = 0.059; Multiparous: Mean = 0.014, SD = 0.031) when considering each channel separately (see Supplementary Table 4).

Third, parent age was negatively correlated with synchrony: *ρ* = −0.11, when considering the average age of parents, and *ρ* = −0.10, p = 0.01, and *ρ* = −0.14, p = 0.001, when considering mothers’ and fathers’ ages respectively (see Supplementary Table 3). Notably, none of the tests was significant on randomly paired, unrelated control dyads.

Additional analyses of parental characteristics in channels which were not significantly different in TOG and SEP conditions could be found in the Supplementary Materials.

## Discussion

Bi-parental rearing intuitively conjures the notion of a coordinated set of responses between co-parenting spouses. The present study investigated whether physical proximity influences brain-to-brain synchrony of co-parents and whether synchrony is being influenced by stimuli and parents’ characteristics. Our first aim was to show that there was greater synchrony when partners were in each other’s physical presence (together-condition; TOG) compared to when they listened to salient vocalisations individually (separate-condition; SEP). We found that synchrony was indeed higher in TOG than SEP in the left inferior frontal gyrus (IFG), left middle frontal gyrus (MFG) and bilateral anterior PFC (aPFC) regions of the brain, all of which fall within the purview of an extensive attentional regulation and cognitive control network^23,24^. The second aim of the study was to prove that the co-presence effect was unique to co-parenting spouses. Our results showed that physical proximity only enhanced synchrony in true couples, but not in control couples, which suggests that synchrony emerged due to the unique presence of one’s spousal partner. From the channels found to be significant, our third aim was to investigate whether acoustic stimuli and parents’ characteristics influenced synchrony. The sounds which induced greater synchrony in TOG than SEP conditions were infant laughter, adult laughter, and static. The fact that positive (i.e. infant and adult laughter) and neutral stimuli (i.e. static), but not negative stimuli (i.e. infant and adult cry), elicited greater synchrony suggests that synchrony depended on the emotional valence of the sound. With regard to parents’ characteristics, synchrony between couples was higher in the left MFG and aPFC when the mother, rather than the father, took the lead in responding to the child. We also found that synchrony was higher in primiparous compared to multiparous couples. Finally, synchrony was also noted to be greater in older parents. Taken together, co-parents showed enhanced brain-to-brain synchrony in the physical proximity of their spousal partner, although this effect was moderated by several stimuli and social factors.

The central finding from this study is that the presence of a spousal partner is associated with greater synchrony in attentional and cognitive control mechanisms. The dorsal and ventral fronto-parietal pathways configure the network that supports attentional regulation functions^27^. Salient human vocalisations have been found to activate these dual attentional pathways^28^. The dorsal stream governs “top-down” (i.e., endogenous) voluntary resources to attend to features or location, whereas the ventral stream oversees resources allocated to “bottom-up” unattended (i.e., exogenous) stimuli that cue attentional shifts. Despite these specialised functions, the two pathways dynamically interact and exert flexible attentional control^29,30^. The MFG acts as a “circuit-breaker” connecting the dorsal and ventral attentional streams^31–33^. In this model, the MFG controls both ventral and dorsal pathways and directs a flexible switch between the “top-down” and “bottom-up” attentional streams^31^ and is involved in involuntary attention shifting^34^. In our study, by displaying a fixation cross “+” that triggered “top-down” attention, followed by its disappearance along with the broadcast of vocalisations that incited “bottom-up” attention, our experimental design specifically targeted at partners’ attentional switching capacities. In this line of reasoning, synchronous activation of the MFG indicates coupled attentional regulation between co-parenting spouses. Moreover, we observed synchronous activation in the left MFG and IFG, areas in the brain that preferentially regulate attention during processing of affective emotional information^25,26^. Besides the MFG and IFG, synchrony also emerged in the bilateral aPFC, which constitutes a component of the frontoparietal control network^24,35,36^. This network is postulated to underpin executive control processes^37^ that promote decision making^38^. In the context of our study, the presence of a spousal partner might facilitate matched executive control processes that could help to organise couples’ upcoming joint behaviours. Our findings are supported by previous studies which have observed co-regulation in couples (e.g.^21,39^). Adopting this perspective, synchrony that emerged in the attentional and cognitive control networks of the brain might reflect couples’ tendency to similarly perceive and process auditory and affective information so as to coordinate joint impending behaviours, when together.

The lack of brain-to-brain synchrony in response to negatively valenced sounds when partners are together might be adaptive for both spousal and co-parenting relationships. Although synchrony is commonly represented as a positive characteristic in the extant literature, synchrony is not necessarily always beneficial for the couple or child. For example, biobehavioural synchrony has also been theorized to indicate poor emotional adjustment^10,40^. In these studies, biophysiological measures, such as cortisol levels^10^ and heart rate variability^40^, exhibited higher synchronous activity in couples who experienced greater conflict and higher levels of stress. There it was theorised that synchrony may be better construed as a tendency for these couples to be more easily affected by each other during stressful periods, therefore reflexively reacting to their partner’s physiological arousal. Likewise, in the context of co-parenting, synchrony in attentional and regulatory mechanisms to stressful cries might indicate maladaptive emotional adjustment between partners that subsequently undermines effective co-parenting. In the real world, cognitive and emotional control allows a parent to attend to a crying child, avoid distractions, and manage impulses and emotions. These capacities support flexible parenting^41^ and are recruited when parents plan and change their behaviours to meet the everyday demands of caregiving^41,42^. If co-parents are prone to being affected by the stress experienced by their partner when their infant is crying, they may not be able to organise effective caregiving behaviours to optimally respond to their child’s needs. Although it is unproven if synchrony is a marker of positive or negative qualities in a spousal relationship, in light of the results from this study we propose that synchronous attentional regulatory mechanisms may generally be adaptive but still become maladaptive in stressful situations.

Parent characteristics, such as frequency of each parent’s response to their child, parental multiparous or primiparous status, and the ages of the parents, were found to moderate the co-presence effect. In general, these variables were only statistically significant in the together condition, which may mean that the presence of the co-parent adds a social dimension to the co-regulation of synchronous responses to external stimuli. First, primiparous parents experienced higher synchrony compared to multiparous parents. Caring for a child is an intensely demanding task that is qualitatively different from other life experiences^43,44^. Primiparous and multiparous mothers follow different parental adjustment trajectories^45^. Primiparous parents may experience a greater need to co-regulate responses than multiparous parents who have had much more experience in caring for their children together. Primiparous parents have greater physiological responses to infant cries than multiparous or even non-parents^46^, suggesting that making decisions relating to child-related cues are indeed of great priority for new parents. Second, a higher frequency of mother (compared to father) taking the lead in responding to the child was related to higher synchrony. Consonant with a family systems perspective, this gender difference may reflect the role of the mother as the primary mediator in the family during stressful situations^47^. As mothers are more likely to employ mediation strategies compared to fathers, they may adapt their responses according to the emotional signals of their partner when co-parenting an infant, and therefore be crucial in determining the level of synchrony in a couple. Last, parental age showed a negative correlation with synchrony, where older couples (or couples with older mothers or older fathers) experienced lower levels of synchrony compared to younger couples. With age comes a greater sense of maturity, competence, and stability^48–52^ suggested that older parents tend to be more secure in their role as parents and have parenting strengths that are consistent with their higher level of maturity. Thus, a lower level of synchrony between older couples may reflect a diminished need to respond similarly to each other as they experience greater security in their own roles as parents. These results point to the malleability of the adult brain during the parenting process and in different phases of life. Thus far, studies have mostly focused on the plasticity of the maternal brain in the context of parenting^53,54^, but it is plausible to deduce that similar brain malleability may be observed in fathers. Unfortunately, the methodology of this study may not be sufficient to definitively conclude the precise direction and magnitude of influence of these social factors on spousal synchrony.

Although the focus of the paper centres on the effect of co-presence of spouses in facilitating synchrony, we have also conducted additional analyses to examine the roles of stimuli- and parent-related factors in channels which did not show a significant difference in synchrony between TOG and SEP. In regard to acoustic stimuli, there was no significant effect of stimuli-related factors in other channels besides the ones which significantly differed in TOG and SEP. These findings suggest a unique role of positively- and neutrally-valenced sounds (i.e. infant laughter, adult laughter, static), but not negatively-valenced sounds (i.e. infant cry, adult cry) in enhancing synchrony in couple’s brain responses in the co-presence of each other. In regard to parent characteristics, higher average co-parenting ratio, primiparous parental status and younger age of parents were all found to be significantly associated with greater synchrony in the TOG condition, even in channels which did not significantly differ between TOG and SEP. These results point to the general effect that parent-related variables have in enhancing synchrony between spouses which might not underpin the difference specifically observed due to the co-presence of a spouse.

Like all research studies, this study carries several limitations. First, we implemented standard infant vocalisations that might not have elicited mother-father brain synchrony as distinctly as own-child vocalisations. Comparing standard to own-child infant vocalisations would have provided a measure of whether synchrony was specific to the child that the couple co-parented. On this limitation, our results may underestimate couple synchrony. Second, we did not include data on parental efficacy and competence. These variables might explain further variance observed in mother-father brain synchrony as related to parenting experience. For example, as children develop, parents could gain a greater sense of competence as individuals or as a couple, which may have implications for couple brain synchrony. Third, our study employed a cross-sectional design, which does not allow us to observe changes in the co-parental brain over time. Different co-parenting partners might exhibit unique changes in the pace and trajectory of brain-to-brain synchrony that might be better captured in a longitudinal design. Fourth, while our sample sample size achieved adequate power to compare between SEP and TOG, future studies ought to employ larger sample sizes that will allow for extensive analyses of the moderating roles of acoustic and social variables. Fifth, we limited the scope of our investigation to the prefrontal cortex. Other brain areas are involved in parental behaviours and responses, including subcortical regions, such as the amygdala^3^. These brain areas may also evince significant couple-specific responses not captured in our study. Sixth, although we included a control static sound, we did not include a baseline reading of brain activation without exposure to any stimuli which might have provided resting state readings of brain synchrony. Finally, our sample consisted of parents with children at heterogenous stages of development, which may be a cause of different responses towards infant vocalisations. For instance, parents with infants may find infant crying and laughter more relevant to their current parenting experience, whereas such vocalisations may not be relevant to the immediate caregiving context of parents with older children. Future studies may opt to disambiguate between sub-populations of parents.

In humans, spousal partners naturally co-parent their infant. While the parental brain has been extensively investigated in regard to infant rearing, research on the influence of co-parenting on caregiving mechanisms is still lacking. Findings from our study suggest that, in the physical presence of a spousal partner, couples exhibit greater brain-to-brain synchrony in regions implicated in attentional regulation and cognitive control. Synchrony is also augmented in response to auditory vocalisations that are positive and neutral, rather than negative. Results from this study can be extrapolated to the real-world context when considering the nature of parenting and spousal relationships. As attentional regulation and cognitive control are especially vital to evoking soothing responses during stressful situations, such as attending to a crying infant^55–57^, matching the brain signals of a stressed spousal partner may be detrimental to co-parenting responses. Conversely, synchrony during favourable situations, such as that invoked by infant laughter, may enhance the spousal relationship. This study presents persuasive evidence that the parental brain may be shaped by the presence of a co-parenting spousal partner, although future studies should investigate how synchrony during positive and negative emotional situations directly affects coordinated caregiving behaviours.

## Materials & Methods

### Participant recruitment

All experimental protocols were approved by the ethics committee of the Psychology Programme of Nanyang Technological University, Singapore. All methods in this study were conducted in accordance with the guidelines and regulations stipulated by the ethical committee. Participants needed to be at least 21 years old to be eligible for this study. 27 pairs of heterosexual couples were recruited through poster and online advertisements on social media platforms, namely Facebook parenting groups and parenting forums. Participants were then screened for their eligibility, with the inclusion criteria as follows: (i) couples who live together; (ii) couples with a young child, aged 4 years or younger; (iii) couples who do not have any known psychological and mental health conditions; and (iv) couples who do not have known medical conditions that affect the oxygen-carrying capacity of their blood. These criteria were selected for their relevance to the experimental procedure. First, couples must live together to afford the opportunity to co-regulate their physiological signals when caring for their child. Second, as experimental stimuli consisted of infant vocalizations (see Table 2), parents with a young child were chosen for their likelihood of being more attentive to these vocalisations^58^. Third, couples should not possess any mental health condition as the study investigates healthy parental populations. Fourth, as fNIRS collects data based on the level of oxygenated and deoxygenated blood within each region of the brain^59^, medical conditions (such as G6PD deficiency^60^) that may cause the data to reflect higher or lower oxygen levels than normal were excluded. Informed consent was obtained from all participants prior to the start of the study. Participants were reimbursed a total of SGD 50 per couple for their time at the end of the study.

**Table 2:** Distress Rating of Low- and High-Pitched Infant Cry.

Stimulus	Gender	Mean	S.D.
Infant cry (low-pitched)	Mothers	2.21	0.885
	Fathers	1.83	0.874
	Combined	1.96	0.876
Infant cry (high-pitched)	Mothers	2.88	1.09
	Fathers	2.29	1.09
	Combined	2.58	1.08

Data were collected from 27 couples (N = 54, Mean age = 33.6, SD = 5.65) consisting of 27 mothers (N = 27, Mean age = 32.8, SD = 5.65) and 27 fathers (N = 27, Mean age = 34.4, SD = 5.67) in two separate experimental conditions: together and separate (see Experimental Procedure), with their youngest child aged 48 months (4 years) and below (N = 27, 15 males and 12 females; Mean age = 17.28 months, SD = 9.43, ranging from 2 to 39 months). We also noted whether the couples were primiparous (one child only) or multiparous (more than one child) as we wanted to investigate whether previous parenting experience affects levels of brain-brain synchrony. Data of 3 couples (n = 6) were excluded from the analysis of the together condition because their children were present in the room (n = 4) or their children cried during the experiment (n = 2), disrupting their attention. The final sample consisted of 24 pairs of parents, of which 10 were primiparous and 14 were multiparous. Data, scripts and materials for this study are available here: 10.21979/N9/KF1JOG and https://gitlab.com/abp-san-public/MF-nirs.

A power analysis (G*Power^61^, version 3.1.9.4, Windows 64 bit) was performed to ensure that the number of couples and stimuli were adequate to detect the effect of being in the TOG condition on the single channel. With an *α* = 0.0025 (corresponding to an *α* = 0.05 with 20 independent tests, i.e. the number of channels), and an average of 260 samples (*N*_*SEP*_ = 130, *N*_*TOG*_ = 130), we are able to detect both strong (d = 0.7) and moderate (d = 0.5) effect sizes with high power (0.99 and 0.83 respectively).

### Questionnaire

Couples completed a questionnaire that recorded their demographic information, parental status (i.e., whether the couple had one or more children), and the age of their youngest child. Each partner was also asked to provide a parenting response ratio; that is, the typical ratio to which the mother:father takes the lead in responding to their child: When you and your partner are together and you hear your child(ren) cry, what is the ratio of Mother:Father taking the lead in attending to the child(ren)? Parents were asked to choose from one of the following options: 0:5, 1:4, 2:3, 3:2, 4:1 and 5:0. The answers were coded into the parent ratio score, from 0 (mother never takes the lead, father always takes the lead) to 1 (mother always takes the lead, father never takes the lead). The scores of the two parents were then averaged to obtain the Average Parent Ratio score.

### Audio Stimuli

Audio stimuli (see Table 3) were selected from online public databases of sound files^62^ to reflect both negative valence sounds, such as crying^63^, and positive valence sounds, such as laughing^64^. A control sound, static noise (Stimulus 6), and adult vocalizations (Stimuli 1 and 5) were included for comparisons to adult and infant sounds.

**Table 3:** Audio Frequency of Experimental Stimuli.

S/No.	Stimulus	Frequency (Hz)
1	Adult female laughter	348.9
2	Infant laughter	331.2
3	Infant cry (low-pitched)	354.3
4	Infant cry (high-pitched)	554.3
5	Adult female cry	318.2
6	Static	Not Applicable

The audio stimuli were analysed for their frequency with Praat software^65–67^ (version 6.1.08, Macintosh 64 bit) to fall within the range of 300 Hz to 400 Hz, with the exception of Stimulus 4, which was modified from Stimulus 3 in terms of frequency to be 200 Hz higher. Stimulus 4, at higher frequency, was intended to induce an enhanced perception of distress^68^, thus providing a means of comparison between relatively less and more distressing infant cries. Table 2 shows the mean and SD of participants’ reported distress on a 5-point scale (1 = Not distressing at all to 5 = Extremely distressing) in response to low- and high-pitched infant cries. Compared to the low-pitched cry, significantly greater distress was reported to the high-pitched cry (t(95) = −4.09, p < 0.05, d = 0.561). All audio stimuli were then digitally equated at 15 s. Stimuli 2, 3, 5, and 6 have been used previously^69^.

NIRStim (NIRx Medical Technologies LLC, 2000, Version 4.0, Windows 64 bit) was used to present all auditory stimuli in a randomised manner. Each stimulus was presented three times (i.e. three trials for each sound) for a duration of 15-s each. A 10-s inter-stimulus interval (ISI) which displayed a white fixation cross “+” against a black background was present between every two stimuli. During the presentation of auditory stimuli, the fixation cross disappeared, and participants were only shown a black screen. The stimuli were screened on a 38-cm Acer Laptop, and participants sat approximately 40 cm in front of the laptop. In the separate-condition, participants wore headphones with volume set at 27.5 dB; in the together-condition, sound stimuli were played aloud with volume set at 44 dB. Earphones were used in the separate-condition to emphasise listening to the sounds in isolation; sounds were played aloud in the together-condition to simulate a shared experience between spousal partners.

### Experimental procedure

**Figure 2:**
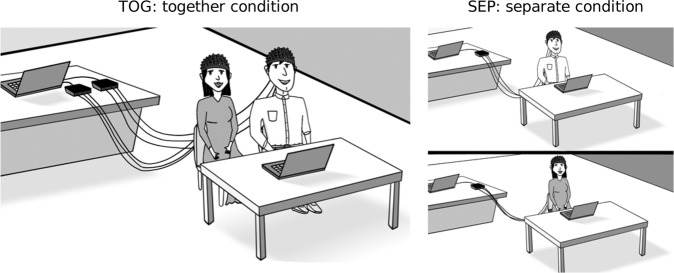
Experimental set-up in together (left) and separated (right) conditions. Figure illustrated by Farouq Azizan.

Couples were invited to attend an experimental session lasting 1.5 hours at the Nanyang Technological University (NTU) Lee Kong Chian School of Medicine (LKCSoM) Campus. Each couple participated in two experimental conditions (see Fig. 2) and order was counterbalanced across couples: (i) together-condition (TOG), in which partners were presented with auditory stimuli at the same time in the same room, and (ii) separate-condition (SEP), in which partners listened to the auditory stimuli in separate rooms and at different times.

The sitting arrangement in the TOG condition was standardised so that the male was always seated on the left and female on the right. Spouses were instructed to refrain from physical contact during the together-condition, as physical touch may result in potentially confounding synchrony^70^. The presence of the fixation cross on the screen assured that participants would not make eye contact with each other during the course of the experiment. Although verbal communication was not restricted during the session, participants were instructed to pay attention to the sounds. The auditory nature of the experimental stimuli enjoined spouses from conversing with each other.

### fNIRS data acquisition

Brain activity of the prefrontal-cortical regions was acquired using the non-invasive fNIRS neuroimaging system (NIRSport, NIRx Medical Technologies LLC), using a sampling rate of 7.81 Hz with light wavelengths at 760 nm and 850 nm^71^. fNIRS allows the quantification of oxygenated and de-oxygenated hemoglobin in different brain areas: brain areas exhibiting higher concentrations of oxygenated haemoglobin (oxy-Hb) indicate localised cerebral activation.

**Figure 3:**
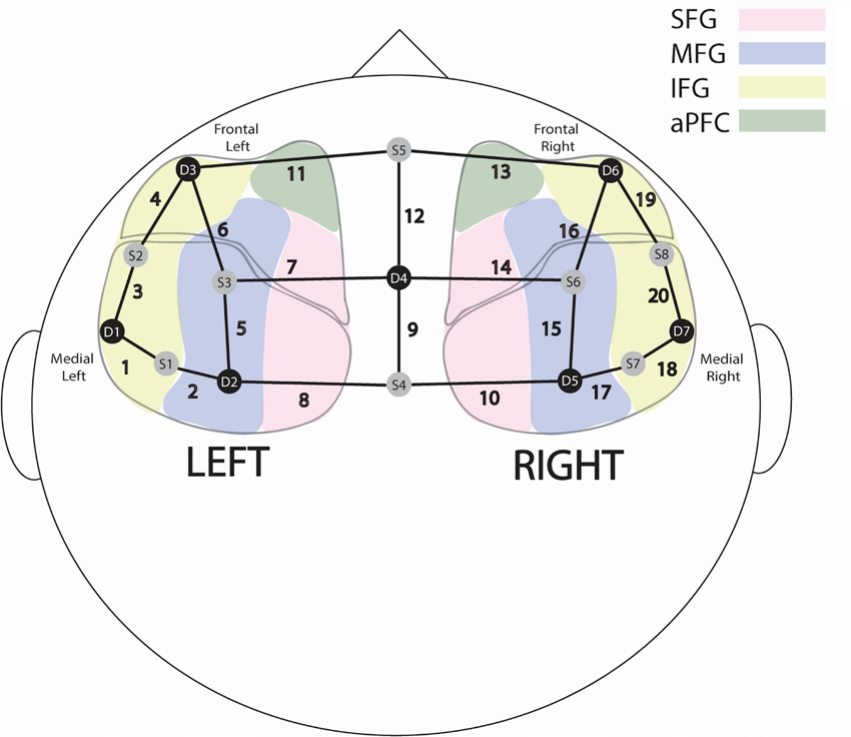
The adopted NIRS montage consisting of 8 sources (gray dots) and 7 detectors (black dots) to form 20 source-detector channels (bold lines). Colors indicate brain areas: Superior Frontal Gyrus (SFG), Middle Frontal Gyrus (MFG), Inferior Frontal Gyrus (IFG) and anterior Pre-Frontal Cortex (aPFC).

The fNIRS cap placed on mothers and fathers utilised a 20-channel system with 8 sources and 7 detectors (Fig. 3). This channel configuration is similar to the international 10–20 system employed in EEG recordings, and analogous brain regions recorded by fNIRS were identified using this system^72^. Previous fNIRS studies have used the same methods of fNIRS channel configuration and brain region analogues^73–75^.

NIRStar software (v15.2, Windows 64 bit) was used to configure the channel setup. During the together-condition the fNIRS data were acquired in tandem hyperscanning mode, and during the separate-condition a single scanning mode was used.

### fNIRS data preprocessing

fNIRS data were first pre-processed using NIRSlab software (NIRx Medical Technologies LLC, v2016.01, Windows 64 bit).

For each subject, channels that presented a gain greater than 8 and coefficient of variation greater than 7.5 were excluded from the analysis, as these characteristics are associated with high signal noise^76–78^. As a consequence of the automatic rejection of channels with high signal noise, a different number of couples was available for analyzing each channel and condition. Spike artifacts, which are signal components with an abnormal change in amplitude, normally produced by head movements, were replaced with the nearest surrounding signals^79^, and discontinuities in the signals, if any, were corrected using the *remove_discontinuities* function on NIRSlab. Finally, a band-pass filter of 0.01–0.2 Hz was applied to eliminate baseline shift variations, and hemoglobin concentrations were determined using the modified Beer-Lambert law. Finally, the signal was visually inspected by two independent experts for validation.

NIRS time-series of oxygenated haemoglobin (oxy-Hb) for each subject, condition, stimulus, and channel were exported from NIRSlab to be analysed.

### Synchrony measures

To obtain an index of synchrony between the brain activities of spousal partners, we computed the similarity in partners’ oxy-Hb concentration levels over time. Cross Correlation, which measures the extent to which two time-series signals co-vary, was used as a time-series similarity metric^80–82^. To account for minimal anticipations or delays of brain activation in one parent with respect to the other, we computed the cross correlation with shifted copies of one signal, between −2 s to 2 s with increments of 0.125 s. We refer to this metric as the Maximum Cross Correlation within a delay of 2 s (MCC2). The same metric has been adopted in the literature to quantify synchrony in different contexts and with different types of physiological signals^80,83–85^. The maximum delay was set to 2 s to account for the temporal characteristics of the brain response signals according to the hemodynamic response function^86^.

For each stimulus, we computed the MCC2 for each trial and then averaged MCC2 data across the three trials as a similarity metric. As a control, we also computed MCC2 between the brain signals of randomly paired mothers and fathers. The random pairing was done for each sound, channel, and condition for all control couples. The computation of synchrony was performed using a custom code based on pyphysio^87^ and physynch packages^85^.

### Analytic plan

The first aim of this study was to show that, compared to individually listening to vocalisations (i.e. SEP condition), the presence of a co-parenting spouse (i.e. TOG condition) would be associated with greater brain-to-brain synchrony between partners. To test this hypothesis, we statistically compared the distributions of TOG and SEP synchrony measures of all stimuli. As we had no prior assumptions about which brain areas should be more affected by the co-presence effect, we tested all 20 channels and corrected the p-values using the False Discovery Rate (FDR) correction with the algorithm proposed by Benjamini and Hochberg^88^ (alpha = 0.05, 20 independent tests, i.e. the number of channels). The two-sided Mann-Whitney test was used to account for the different number of couples in the two conditions.

Our second aim was to confirm that the co-presence effect was unique to the presence of a spouse (i.e. true couples). Thus, we randomly paired the brain signals of mothers and fathers who were not couples and generated synchrony indexes for the control dyads. We then compared the distributions of the TOG and SEP synchrony measures for control dyads.

Our third aim was more exploratory in nature, namely to investigate whether the emotional valence of sound stimuli (i.e. positive vs negative vocalisations) and parents’ characteristics, (i) ratio of mother to father taking the lead in attending to infant, (ii) primiparous compared to multiparous parents and (iii) parents’ age, influenced the extent of synchrony. For each channel in which the co-presence of a spouse significantly led to greater synchrony, we compared (Mann-Whitney test, one-sided) the synchrony index for each sound stimulus in both SEP and TOG conditions, and corrected the p-values (Benjamini-Hochberg FDR^88^, alpha = 0.05, 6 independent tests, i.e. the number of stimuli). As for the social variables, we compared primiparous and multiparous couples (Mann-Whitney test, two-sided) and calculated Spearman correlations to investigate the influence of the Average Parent Ratio (i.e. ratio of mother to father taking the lead in responding to the child) and age of parents. Each statistical test was first performed on the data of all channels together then separately on each of the channels which resulted significant in the SEP v. TOG analysis. P-values of the tests on the separate channels were corrected for multiple hypotheses (Benjamini-Hochberg FDR^88^, alpha = 0.05, 4 independent tests, i.e. four channels). All tests were also performed on the randomly paired dyads as a control.

## Supplementary information

Supplementary Information
