# Supplementary material for: Physical presence of spouse enhances brain-to-brain synchrony in co-parenting couples
Source: Sci Rep. 2020 May 5;10:7569. doi: 10.1038/s41598-020-63596-2 (PMC7200679; doi:10.1038/s41598-020-63596-2)
Supplement: Supplementary file 1 — Supplementary Information [file 41598_2020_63596_MOESM1_ESM.pdf]

Physical presence of spouse enhances brain-to-brain synchrony in co-parenting couples

Atiqah Azhari[1], Mengyu Lim[1], Andrea Bizzego[2], Giulio Gabrieli[1], Marc H. Bornstein[3], and Gianluca Esposito[1,2,*]

[1]Psychology Program, School of Social Sciences, Nanyang Technological University, Singapore
[2]Division of Psychology, Department of Psychology and Cognitive Science, University of Trento, Italy
[3]National Institute of Child Health and Human Development, USA, and Institute for Fiscal Studies, United Kingdom
[*]gianluca.esposito@ntu.edu.sg, gianluca.esposito@unitn.it

ABSTRACT

Co-parenting spouses who live together remain in close physical proximity to each other and regularly engage in reciprocal social interactions in joint endeavors to coordinate their caregiving. Although bi-parental rearing is a common occurrence in humans, the influence of the physical presence of a co-parenting spouse on parental brain responses remains largely unknown. Synchrony is conceptualized as the matching of behavioral and physiological signals between two individuals. In this study, we examined how the presence of a co-parenting spouse influences brain-to-brain synchrony when attending to salient infant and adult vocalizations. We hypothesized that brain-to-brain synchrony would be greater in the presence of a spousal partner. Functional Near-infrared Spectroscopy (fNIRS) was used on 24 mother-father dyads (N = 48) to measure prefrontal cortical (PFC) activities while they listened to infant and adult vocalizations in two conditions, together (in the same room at the same time) and separately (in different rooms at different times). Couples showed greater synchrony in the together condition; when comparing fNIRS data between true couples and randomly matched controls, this synchronous effect was only seen in true couples, indicating a unique effect of spousal co-regulation toward salient stimuli. Our results indicate that the physical presence of the spouse might establish synchrony in attentional regulation mechanisms toward socially relevant stimuli. This finding holds implications for the role of the co-parenting spouse in influencing social and parental brain mechanisms.

Supplementary Information

Results

channel	Mean_SEP	SD_SEP	N_SEP	Mean_TOG	SD_TOG	N_TOG	p (uncorrected)
1	0.006	0.046	135	0.033	0.107	132	0.02842
2	0.021	0.048	141	0.047	0.105	126	0.09134
3	0.007	0.025	141	0.031	0.083	138	0.00221
4	0.015	0.051	141	0.041	0.095	120	0.02187
5	0.040	0.178	141	0.021	0.300	138	0.54731
6	0.010	0.035	141	0.016	0.039	132	0.13982
7	0.009	0.025	135	0.024	0.045	132	0.00041
8	0.024	0.064	135	0.045	0.087	132	0.18962
9	0.015	0.054	141	0.037	0.116	138	0.09633
10	0.011	0.047	135	0.031	0.065	126	0.04381
11	0.013	0.064	135	0.062	0.119	138	0.00003
12	0.010	0.029	135	0.021	0.039	138	0.03754
13	0.026	0.109	135	0.043	0.090	138	0.00484
14	0.008	0.019	141	0.012	0.024	132	0.34118
15	0.039	0.168	135	0.021	0.118	138	0.73530
16	0.001	0.362	135	0.022	0.060	138	0.03153
17	0.019	0.050	135	0.095	0.291	126	0.34332
18	0.011	0.034	141	0.020	0.046	138	0.49809
19	0.006	0.069	135	0.023	0.048	126	0.09338
20	0.012	0.034	141	0.024	0.085	120	0.41871

Table 1. Results of the comparison between SEP v. TOG conditions for all fNIRS channels.

	Average Parent Ratio			
	SEP		TOG	
Channels	rho	p-value	rho	p-value
all	0.00	0.97430	0.18	0.00003
3	0.01	0.89425	0.06	0.47776[a]
7	0.07	0.80057	0.29	0.00355[a]
11	-0.07	0.80057	0.23	0.01515[a]
13	0.04	0.83270	0.18	0.05322[a]

Table 2. Correlations between the mother-father synchrony and the Average Parenting Ratio score.
[a]: corrected for multiple hypothesis (FDR B-H Method, 4 independent tests, i.e. the four channels)

	Average Parent Age				Mother Age				Father Age			
	SEP		TOG		SEP		TOG		SEP		TOG	
Channels	rho	p-value	rho	p-value	rho	p-value	rho	p-value	rho	p-value	rho	p-value
all	0.03	0.43649	-0.11	0.01039	0.02	0.68256	-0.10	0.01533	0.04	0.30656	-0.14	0.00135
3	0.03	0.89992[a]	-0.04	0.63450[a]	-0.02	0.91935[a]	-0.01	0.90244[a]	0.12	0.69690[a]	-0.11	0.27955[a]
7	0.04	0.89992[a]	-0.07	0.58192[a]	0.03	0.91935[a]	-0.10	0.32364[a]	0.02	0.84337[a]	-0.03	0.73314[a]
11	0.07	0.89992[a]	-0.13	0.26535[a]	0.06	0.91935[a]	-0.11	0.32364[a]	0.08	0.76691[a]	-0.17	0.08055[a]
13	-0.01	0.89992[a]	-0.21	0.05693[a]	-0.01	0.91935[a]	-0.20	0.07297[a]	-0.04	0.84337[a]	-0.22	0.03201[a]

Table 3. Correlations between the mother-father synchrony and age of parent.
[a]: corrected for multiple hypothesis (FDR B-H Method, 4 independent tests, i.e. the four channels)

	SEP					TOG				
	Primiparous			Multiparous		Primiparous			Multiparous	
Channels	Mean	SD	p-value	Mean	SD	Mean	SD	p-value	Mean	SD
all	0.017	0.094	0.45326	0.011	0.034	0.060	0.117	0.00064	0.028	0.065
3	0.003	0.023	0.30659[a]	0.010	0.027	0.036	0.089	0.31024[a]	0.027	0.079
7	0.010	0.033	0.44420[a]	0.008	0.018	0.041	0.059	0.00351[a]	0.014	0.031
11	0.009	0.082	0.44420[a]	0.016	0.046	0.094	0.166	0.09161[a]	0.042	0.069
13	0.045	0.161	0.30659[a]	0.012	0.037	0.067	0.114	0.06855[a]	0.028	0.067

Table 4. Results of the comparison of the mother-father synchrony between Primiparous and Multiparous parents, in the two experimental conditions.
[a]: corrected for multiple hypothesis (FDR B-H Method, 4 independent tests, i.e. the four channels)
